# Oxynitride-surface engineering of rhodium-decorated gallium nitride for efficient thermocatalytic hydrogenation of carbon dioxide to carbon monoxide

**DOI:** 10.1038/s42004-022-00728-x

**Published:** 2022-09-06

**Authors:** Jinglin Li, Bowen Sheng, Yiqing Chen, Sharif Md. Sadaf, Jiajia Yang, Ping Wang, Hu Pan, Tao Ma, Lei Zhu, Jun Song, He Lin, Xinqiang Wang, Zhen Huang, Baowen Zhou

**Affiliations:** 1 Key Laboratory for Power Machinery and Engineering of Ministry of Education, School of Mechanical Engineering, Shanghai Jiao Tong University, 800 Dongchuan Road, Shanghai, 200240 China; 2 State Key Laboratory of Artificial Microstructure and Mesoscopic Physics, School of Physics, Nano-Optoelectronics Frontier Center of Ministry of Education (NFC-MOE), Peking University, Beijing, 10087 China; 3 Department of Mining and Materials Engineering, McGill University, 3610 University Street, Montreal, QC H3A0C9 Canada; 4 Centre Energie, Matériaux et Télécommunications, Institut National de la Recherche Scientifique (INRS)-Université du Québec, 1650 Boulevard Lionel-Boulet, Varennes, QC J3X1S2 Canada; 5 Michigan Center for Materials and Characterization, University of Michigan, 2800 Plymouth Rd, Ann Arbor, MI 48109 USA; 6 Peking University Yangtze Delta Institute of Optoelectronics, Nantong, Jiangsu 226010 China; 7 Collaborative Innovation Center of Quantum Matter, School of Physics, Peking University, Beijing, 100871 China

**Keywords:** Heterogeneous catalysis, Thermoelectrics, Nanowires, Nanoparticles

## Abstract

Upcycling of carbon dioxide towards fuels and value-added chemicals poses an opportunity to overcome challenges faced by depleting fossil fuels and climate change. Herein, combining highly controllable molecular beam epitaxy growth of gallium nitride (GaN) under a nitrogen-rich atmosphere with subsequent air annealing, a tunable platform of gallium oxynitride (GaN_1-*x*_O_*x*_) nanowires is built to anchor rhodium (Rh) nanoparticles for carbon dioxide hydrogenation. By correlatively employing various spectroscopic and microscopic characterizations, as well as density functional theory calculations, it is revealed that the engineered oxynitride surface of GaN works in synergy with Rh to achieve a dramatically reduced energy barrier. Meanwhile, the potential-determining step is switched from *COOH formation into *CO desorption. As a result, significantly improved CO activity of 127 mmol‧g_cat_^−1^‧h^−1^ is achieved with high selectivity of >94% at 290 °C under atmospheric pressure, which is three orders of magnitude higher than that of commercial Rh/Al_2_O_3_. Furthermore, capitalizing on the high dispersion of the Rh species, the architecture illustrates a decent turnover frequency of 270 mol CO per mol Rh per hour over 9 cycles of operation. This work presents a viable strategy for promoting CO_2_ refining via surface engineering of an advanced support, in collaboration with a suitable metal cocatalyst.

## Abstract (web summary)

Thermocatalytic hydrogenation holds great promise for commercial utilization of carbon dioxide, but the process is energy-intense with high temperature and pressure requirements. Here, the authors engineer a GaN_1-__*x*_O_*x*_ rhodium nanoparticle catalyst for CO_2_ to CO hydrogenation that functions at temperatures as low as 170 °C.

## Introduction

The trillions of tons of anthropogenic CO_2_ emitted into the atmosphere appear as one of the most significant concerns in the 21st century^1–4^. It is highly urgent to address this critical issue at the lowest expense. Compared to carbon dioxide capture and sequestration (CCS) technology with potential ecology risk, recycling CO_2_ into fuels and chemicals provides an ultimate solution for the ever-growing global energy demand and destructive climate change^5,6^. As opposed to photocatalysis, electrocatalysis and bio-catalysis, owing to its huge processing capacity and high compatibility with the existing chemical industry^7^, thermocatalytic hydrogenation holds great promise for commercial utilization of carbon dioxide. However, thermocatalytic CO_2_ hydrogenation suffers from high temperature and high pressure, which is energy-intensive. From the viewpoint of sustainability, it is greatly desired to explore relatively mild strategies for CO_2_ hydrogenation toward fuels and chemicals but remains a grand challenge.

CO, a component of syngas, is the key feedstock for producing enormous synthetic fuels and chemical commodities^8–11^. Compared to high-order products, e.g., alcohols, alkanes, and olefins via deep hydrogenation, the production of CO from CO_2_ hydrogenation is both kinetically and thermodynamically favored. However, CO_2_ hydrogenation to CO, namely reverse water gas shift (RWGS) reaction, is a typical endothermic process^12,13^. Because of the inert nature of CO_2_ (linear C=O bond, 803 kJ/mol)^14^ and intricate reaction network, highly efficient and selective production of the desired product from CO_2_ hydrogenation remains a grand challenge, which is, in principle, limited by sluggish kinetics and high energy barriers^8,15^. Over the past decades, although various catalysts have been developed for CO_2_ hydrogenation, the performance is yet far from the practical level, which is largely due to lack of a suitable support. For instance, Rh metal is well known for CO_2_ hydrogenation because of its unique catalytic properties^6,16–18^. However, the most used metal oxides support, e.g., Al_2_O_3_ and CeO_2_ did not show obvious synergy with Rh species for CO_2_ hydrogenation, thus suffering from limited activity^19–21^. If great support in which the surface property could be facilely engineered for immobilizing a suitable catalyst to synergistically reduce the reaction energy barrier, it may be greatly promising for CO_2_ hydrogenation.

Industry ready, group III-nitrides materials system with widespread applications in power electronics, photonic devices, and massive solid lighting^22–24^, has attracted little interest in CO_2_ refining despite the distinct structural, optical and electronic properties. In the pioneering attempts^25–28^, nanostructured III-nitrides, e.g., GaN were highly promising to immobilize metal sites for photocatalytic CO_2_ reduction due to the following reasons: (i) Firstly, one-dimensional (1D) morphology and high surface area enable high dispersion of active sites; (ii) Secondly, the wurtzite GaN can be directly involved in activating the linear non-polar CO_2_ molecules; (iii) Most importantly, the surface of GaN grown under N-rich atmosphere could be engineered at atomic scale for mediating the catalytic behavior; which is essentially distinct from the conventional supports. Collectively, the 1D nanostructured GaN presents a revolutionary platform for immobilizing a suitable cocatalyst to assemble a rational catalytic architecture.

In this work, we have achieved the great CO_2_ hydrogenation towards CO efficiency by combining the tunable platform of GaN_1–*x*_O_*x*_ after surface engineering with Rh NPs. Both experimental and theoretical studies reveal that the oxynitride surface of GaN_1–*x*_O_*x*_ works in synergy with the immobilized Rh NPs to achieve a significantly reduced activation barrier by switching the potential-determining step from *COOH formation toward *CO desorption. Under the optimized conditions, the reaction could be initiated at a temperature as low as 170 °C; and a high CO activity of 127 mmol·g_cat_^−1^·h^−1^ with high selectivity of >94% is achieved at 290 °C under ambient pressure, which clearly outperforms the commercial catalyst of Rh/Al_2_O_3_. Moreover, it is superior to most of the state-of-the-art catalysts reported (Table S1). Benefitting from the high dispersion of Rh species, a high turnover frequency (TOF) of 270 mol CO per mol Rh per hour is realized, resulting in total turnover number (TON) of 2616 mol CO per mol Rh after 9 cycles operation. To our best knowledge, this study presents the first endeavor in utilization and surface oxidation of GaN to coupling with suitable active sites for CO_2_ hydrogenation by thermocatalysis.

## Results and discussion

### Assembly and characterization of the architecture

**Fig. 1:**
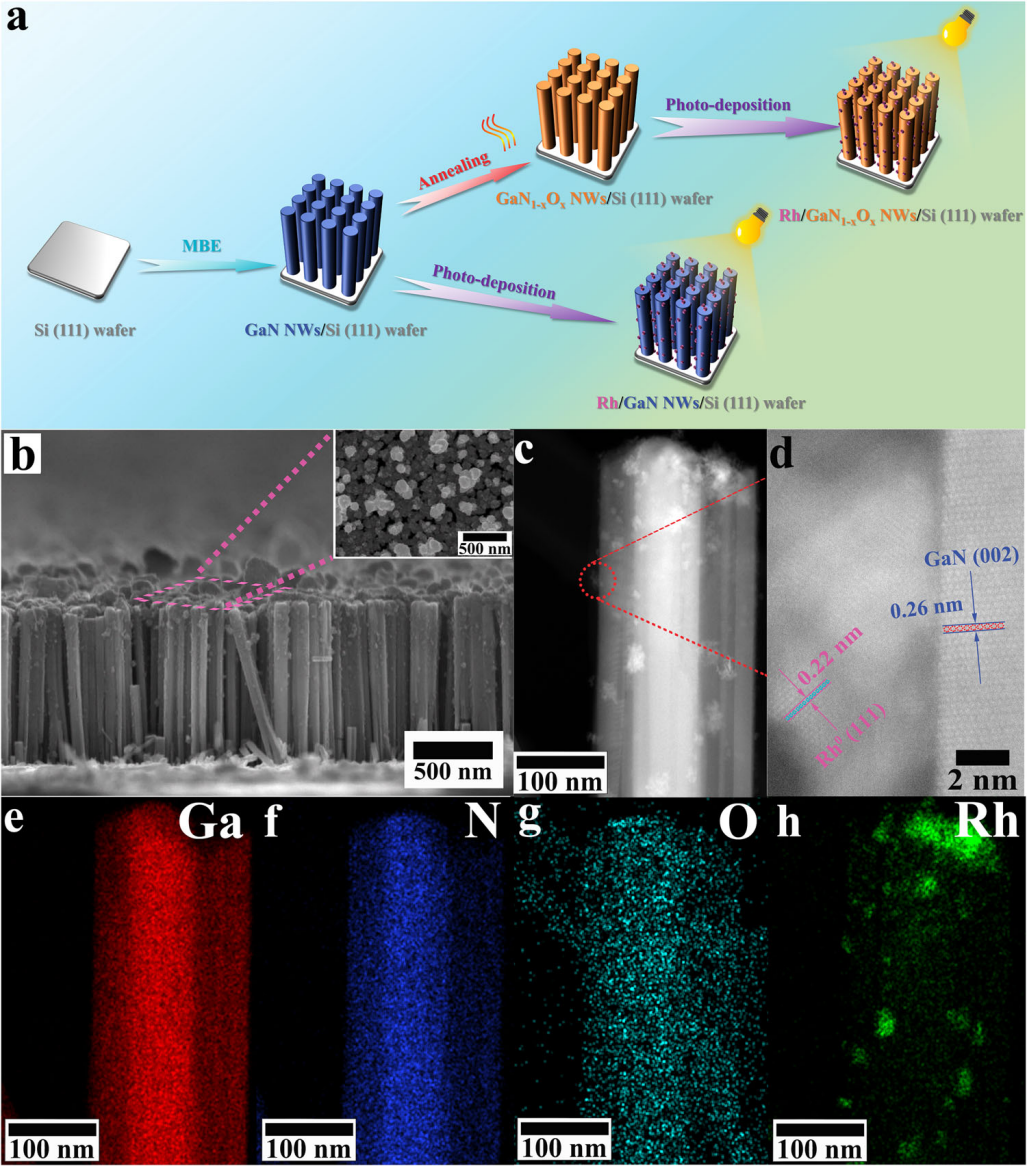
Assembly process and microstructure of catalysts. **a** Schematic diagram for the decoration of Rh NPs onto GaN NWs by combining MBE with photo-deposition. **b** SEM image of Rh NPs supported by GaN NWs that vertically aligned on silicon (111) wafer. **c** HAADF-STEM and **d** HR-STEM images of Rh NPs supported by GaN NW. **e**–**h** EDS mapping of Rh/GaN.

By employing state-of-the-art molecular beam epitaxy technique, GaN nanowires (NWs) were first vertically grown onto a 4-inch Si (111) wafer under N-rich atmosphere (Fig. 1a). As characterized by the scanning electron microscopy (SEM), the epitaxial GaN NWs feature a length in the range of 600–900 nm with diameters in the range of 50–100 nm (Fig. 1b and Fig. S1). The well-defined 1D morphology and high surface area make GaN NWs suitable ideal scaffolds for anchoring catalytic sites. Subsequently, upon annealing in air under various temperatures (150, 200, and 250 °C), GaN was engineered into gallium oxynitride (GaN_1–*x*_O_*x*_) by partially replacing N atoms with O atoms, which will be studied by X-ray photoelectron spectroscopy measurement. The samples annealed at various temperatures in air were denoted as GaN_1–*x*_O_*x*_-150, GaN_1–*x*_O_*x*_-200, and GaN_1–*x*_O_*x*_-250, according to the annealing temperatures of 150, 200, and 250 °C. In contrast with the conventional supports, e.g., Al_2_O_3_, the annealed NWs may offer a tunable platform for modulating the catalytic properties of the anchored active sites, thus mediating CO_2_ hydrogenation. After photo-deposition, as shown in high-angle annular dark-field scanning transmission electron microscopy (HAADF-STEM) images (Fig. 1c, d), Rh NPs with a size of 9.1–11.8 nm were randomly distributed on the surface of GaN (Fig. S2). Even after annealing at 250 °C for 1 h, no significant changes in the size of Rh NPs (8.5–11.3 nm) and 1D morphology of the support were observed compared to that of no annealing (Fig. S3). The lattice spacing of 0.26 nm attributes to the (002) plane of GaN, implying that the growth direction of nanowires is the c-axis, in agreement with the XRD patterns (Fig. S4). The lattice spacing of 0.22 nm is assigned to the (111) plane of metallic Rh^29,30^. Note that no diffraction peaks of Rh were observed by XRD, possibly due to its low concentration (0.047 μmol·cm^−2^ determined by inductively coupled plasma-atomic emission spectroscopy (ICP- AES)). The energy dispersive X-ray spectroscopy (EDS) mapping further confirmed the presence of Rh NPs immobilized by the GaN scaffold (Fig. 1e–h).

**Fig. 2:**
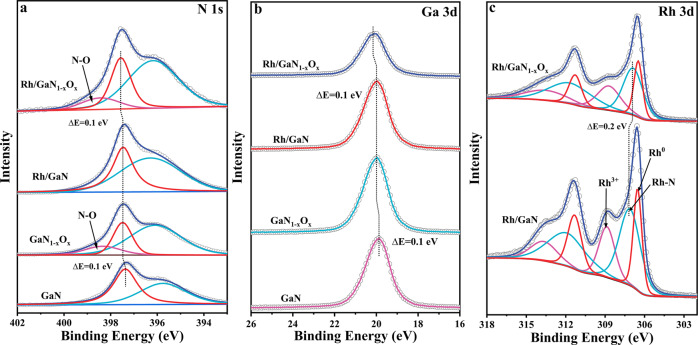
X-ray photoelectron spectroscopy analysis of as-prepared samples. High-resolution XPS spectra of N 1s (**a**), Ga 3d (**b**) and Rh 3d (**c**) for GaN, GaN_1–*x*_O_*x*_, Rh/GaN, and Rh/GaN_1–*x*_O_*x*_.

Considering that the surface property of the support may affect the reaction significantly, the as-prepared architectures were deeply studied by X-ray photoelectron spectroscopy (XPS) (Fig. S5). Due to the N-rich growth conditions of molecular beam epitaxy, there were no nitrogen vacancies observed for the assembled architecture. One noticeable observation is that compared to the typical peak of N 1s appearing at 397.4 eV over the fresh GaN, a featured peak of oxynitride (N-O) at around 399.9 eV was clearly observed for GaN_1–*x*_O_*x*_ (Fig. 2a)^26^. This discovery provides direct evidence that upon annealing under air atmosphere, the N-rich surface of wurtzite GaN could be engineered into oxynitride. It indeed affects the catalytic properties of the architecture as studied by spectroscopic measurements and theoretical calculations in the following. Figure S6 demonstrated that the HR-XPS integral area of O 1s increased with the increasing annealing temperature. The finding suggested that the oxidation degree was positively correlated with the annealing temperature. Meanwhile, the typical peaks of O 1s continued to shift to lower binding energies as the annealing temperature increased. What is more, the binding energies of both N 1s and Ga 3d in Rh/GaN and Rh/GaN_1–*x*_O_*x*_ exhibited obvious shifts compared to that of bare GaN and GaN_1–*x*_O_*x*_, correspondingly, suggesting the strong interaction between the platforms and cocatalyst (Fig. 2a, b)^26,31^, which is beneficial for their synergy to promote the reaction. Upon decoration with cocatalyst, HR-XPS measurement confirmed the coexistence of Rh^0^ (~306.5 eV) and Rh^3+^ (308.8 eV) in the immobilized Rh species (Fig. 2c)^30^. It is worth noting that, the feature peaks of Rh 3d in Rh/GaN_1–*x*_O_*x*_ shifted by 0.2 eV compared to that Rh/GaN, further evidencing that t the surface engineering of GaN affected the electronic properties of Rh species, thus affecting CO_2_ hydrogenation. The afore-discussed results verify the successful engineering of the surface property of GaN to immobilize Rh species for CO_2_ hydrogenation.

### Catalytic CO_2_ hydrogenation

**Fig. 3:**
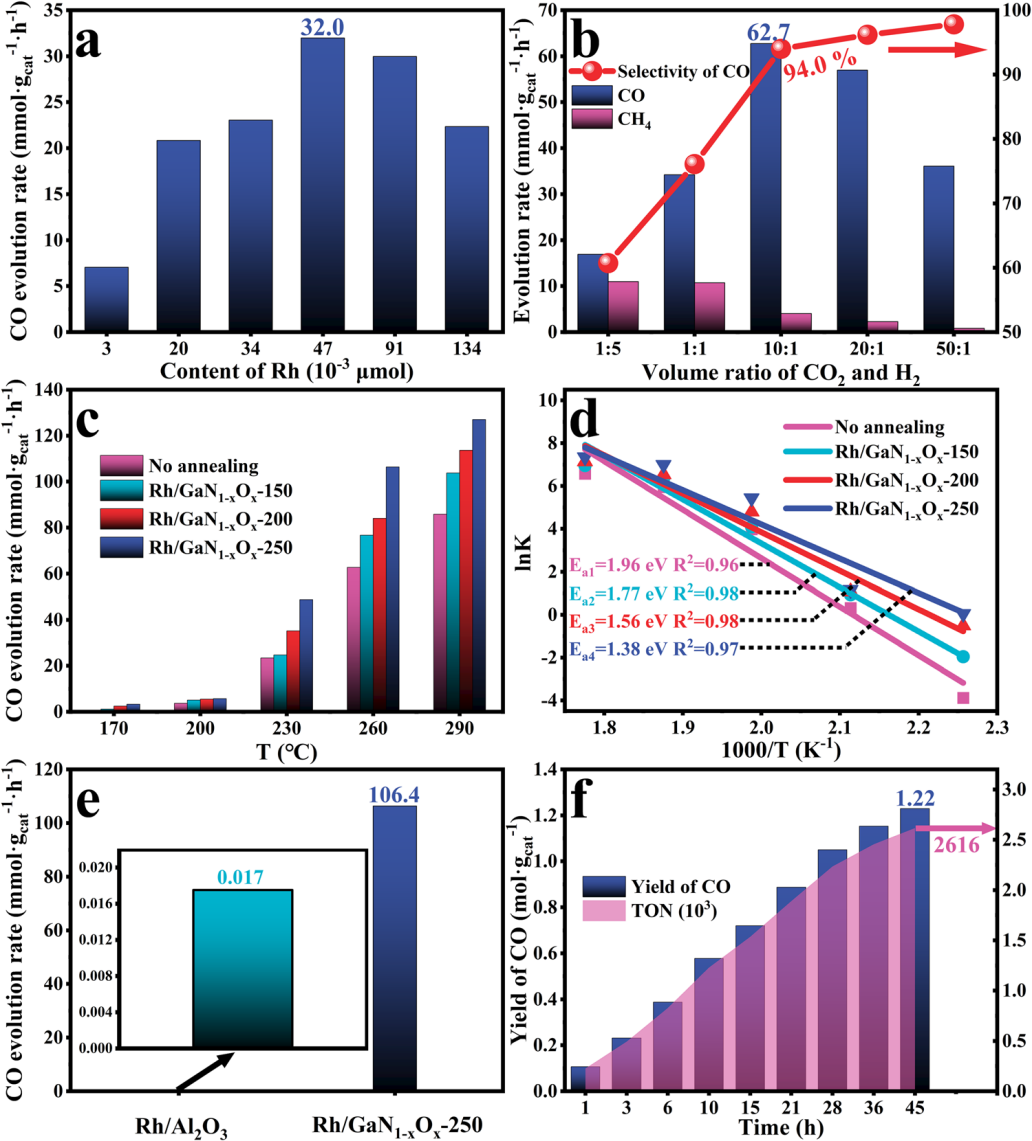
Thermo-catalytic CO_2_ hydrogenation. Influence of the loaded content of Rh (**a**) and CO_2_/H_2_ ratios (**b**) on the performance. **c** CO evolution rate of Rh/GaN and various annealed Rh/GaN_1*–x*_O_*x*_ samples. **d** Apparent activation barrier (*E*_*a*_) for CO_2_ hydrogenation on Rh/GaN and various annealed Rh/GaN_1–*x*_O_*x*_ samples. **e** CO activity comparison between Rh/GaN_1–*x*_O_*x*_-250 and commercial Rh/Al_2_O_3_ catalyst at 260 °C. **f** CO yield and TON over Rh/GaN_1–*x*_O_*x*_-250 after 9 cycles operation.

The performance of CO_2_ hydrogenation of various catalytic architectures was evaluated in a closed stainless-steel chamber under atmospheric pressure; and the results are shown in Fig. 3. By varying the volume of Rh precursor (Na_3_RhCl_6_‧XH_2_O), the influence of the loaded content of Rh on the reaction was first investigated (Fig. 3a). At low content of Rh of 0.003 µmol·cm^−2^, the architecture showed a slight CO evolution rate of 7.0 mmol·g_cat_^−1^·h^−1^ because of the deficient catalytic sites (Fig. S7a, b). The activity of CO was increased considerably with the increasing content of Rh species; and peaked with a rate of 32.0 mmol·g_cat_^−1^·h^−1^ at a higher Rh content of 0.047 μmol·cm^−2^, as a result of sufficient catalytic sites with appropriate size^32^. However, overloading of Rh gave rise to a decreased activity of 22.3 mmol·g_cat_^−1^·h^−1^, which is probably attributed to severe particle agglomeration and poor dispersion (Fig. S7c, d). Furthermore, the CO activity and selectivity of the designed catalyst is highly sensitive to the feedstock. As illustrated in Fig. 3b, a volcano trend of CO activity was demonstrated by varying the CO_2_/H_2_ ratios. In particular, a mild CO activity of 16.9 mmol·g_cat_^−1^·h^−1^ was obtained when CO_2_/H_2_ ratio was set at 1/5. In this case, the selectivity of CO is as low as 60.7%, in concurrent formation of CH_4_ as a major byproduct. A higher CO_2_/H_2_ ratio of 10/1 enabled an optimal CO activity of 62.7 mmol·g_cat_^−1^·h^−1^ with superior selectivity of 94%. This phenomenon is due to that the hydrogen coverage of the catalysts surface was improved when the hydrogen concentration got risen, which facilitated the deep hydrogenation of CO_2_ toward CH_4_ but leaded to the reduction of CO_2_ conversion ratio. Further increment in CO_2_/H_2_ ratio resulted in a decreased activity despite the enhanced CO selectivity, owing to the lack of sufficient hydrogen for driving the reaction.

As expected, as displayed in Fig. 3c, the performance of the catalysts is very sensitive to the reaction temperature. Particularly, compared to the fresh Rh/GaN, the annealed sample is even able to trigger the reaction at a low temperature of 170  °C. It is further discovered that the enhancement in CO activity was positively correlated with the annealing temperature. A high activity of 127.0 mmol·g_cat_^−1^·h^−1^ was achieved at 290 °C under atmospheric pressure over the optimized Rh/GaN_1-*x*_O_*x*_−250, which is 47.8% higher than that of 85.9 mmol·g_cat_^−1^·h^−1^ over Rh/GaN. However, higher annealing temperatures resulted in degraded activity (Fig. S8). It was attributed to the excessive surface oxidation, which led to inefficient adsorption of CO_2_. Meanwhile, for the catalysts tested, they all exhibited superior selectivity of higher than 94% over the reaction temperature ranged from 170 to 290 °C (Fig. S9). By comparison, CO activity of Rh/GaN_1–*x*_O_*x*_-250 reached 106.4 mmol·g_cat_^−1^·h^−1^ at 260 °C (Fig. 3e), which is 6259 times higher than that of the commercial catalyst of Rh/Al_2_O_3_ (0.017 mmol·g_cat_^−1^·h^−1^) under the same condition (The morphology and elemental characterization of the commercial Rh/Al_2_O_3_ were shown in Fig. S10). In addition, the performance of Rh/GaN_1–*x*_O_*x*_-250 is even superior to state-of-the-art catalysts that operate under higher temperature and more elevated pressure (Table S1), evidencing the feasibility of engineering the support’s surface properties to promote carbon dioxide hydrogenation. Benefitting from the low content of Rh species with high dispersion, a decent turnover frequency (TOF) of 270.2 mol CO per mol Rh per hour was achieved under the relatively moderate conditions (Fig. S11). After nine cycles of hydrogenation, the architecture of Rh/GaN_1–*x*_O_*x*_-250 has enabled the achievement of a high total turnover number of 2616 mol CO per mol Rh without obvious degradation (Fig. 3f), validating the appreciable stability. However, after that, the agglomeration of Rh NPs was observed by TEM (Fig. S12), explaining the performance degradation after 45 h testing. To essentially describe the promotion effect, the apparent activation barrier (*E*_a_) of CO_2_ hydrogenation toward CO was calculated (Fig. 3d). Without annealing, the *E*_a_ of Rh/GaN was determined to be as high as 1.96 eV, suggesting limited activation of the reactants. As a notable contrast, the activation barriers of Rh/GaN_1-*x*_O_*x*_-150 and Rh/GaN_1-*x*_O_*x*_-200 decreased to 1.77 and 1.56 eV, respectively. Notably, a dramatic reduction in *E*_*a*_ downward to 1.38 eV was obtained for Rh/GaN_1–*x*_O_*x*_-250 by increasing the annealing temperature to 250 °C. These results provide solid evidence that the surface engineering of GaN via straightforward annealing can promote CO_2_ hydrogenation by reducing the activation barrier. As a comparison, the commercial GaN thin film-supported Rh species was also found to illustrate catalytic activity for CO_2_ hydrogenation toward CO (Figs. S13 and S14) although the activity was relatively low. However, there was no observation of CO yielded from CO_2_ on Rh/Si in the absence of GaN. This result validated the critical role of GaN in the reaction.

### Origin of the improved performance from the surface engineering of GaN

**Fig. 4:**
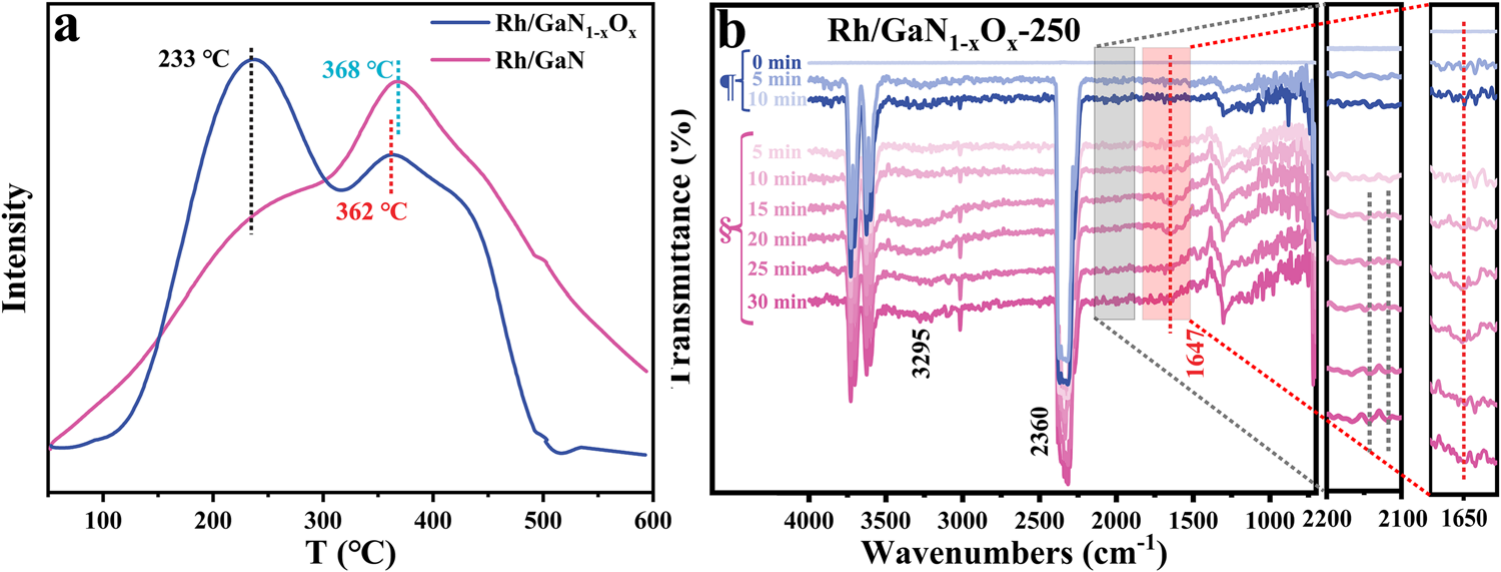
Spectroscopic analysis of catalytic mechanism. The desorption behavior of **a** CO over Rh/GaN and Rh/GaN_1–*x*_O_*x*_-250 by TPD. **b** In situ DRIFT spectra of Rh/GaN_1–*x*_O_*x*_-250, ¶: from room temperature to 260 °C; §: keeping 260 °C.

Temperature programmed desorption (TPD) technique, in combination with in situ diffuse reflectance infrared Fourier-transform spectroscopy (DRIFTS) characterization and density functional theory (DFT) calculations, were correlatively employed to study the promotion effect. The desorption behavior of CO was first investigated by TPD technique. As shown in Fig. 4a, the desorption temperature of CO from Rh/GaN_1–*x*_O_*x*_-250 is as low as 233 °C, which is much lower than that of Rh/GaN (368 °C), suggesting the facile desorption of CO from the interface of Rh/GaN_1–*x*_O_*x*_-250 once it is formed. This finding is well consistent with the measured activity and theoretical calculations.

In situ DRIFTS characterization was further conducted to study the evolution of CO from CO_2_. As shown in Fig. 4b, the typical peaks at around 2360 cm^−1^ are associated with the characteristic rotation vibration of CO_2_^33–35^, indicating facile adsorption of CO_2_ over either Rh/GaN or Rh/GaN_1–*x*_O_*x*_ interface at low temperature. Upon external heating, the featured peak appeared at 1647 cm^−1^ could be attributed to *COOH, suggesting that *COOH was likely to be the key intermediate of the reaction^36–38^. Moreover, the typical peak emerging at around 3295 cm^−1^ was related to the vibration bands of υ(OH) in the adsorbed H_2_O^36^, indicating the formation of H_2_O from CO_2_ hydrogenation. Another relatively weak peak located between 2100 and 2200 cm^−1^ were related to gaseous or absorbed CO intermediate^33^. Herein, Rh/GaN showed a DRIFT spectrum similar to Rh/GaN_1–*x*_O_*x*_ despite the varied intensity of the typical features (Fig. S15a). Moreover, the strengthened intensity of the typical peaks of *COOH and H_2_O suggested the accumulation of the two species as the reaction proceeded. In addition, compared to Rh/GaN, relatively high intensities of key intermediate of *COOH can be observed for the annealed sample during the entire process examined, suggesting the promotion effect of the surface engineering on producing the key intermediate during CO_2_ hydrogenation (Fig. S15b), resulting in dramatically enhanced activity.

**Fig. 5:**
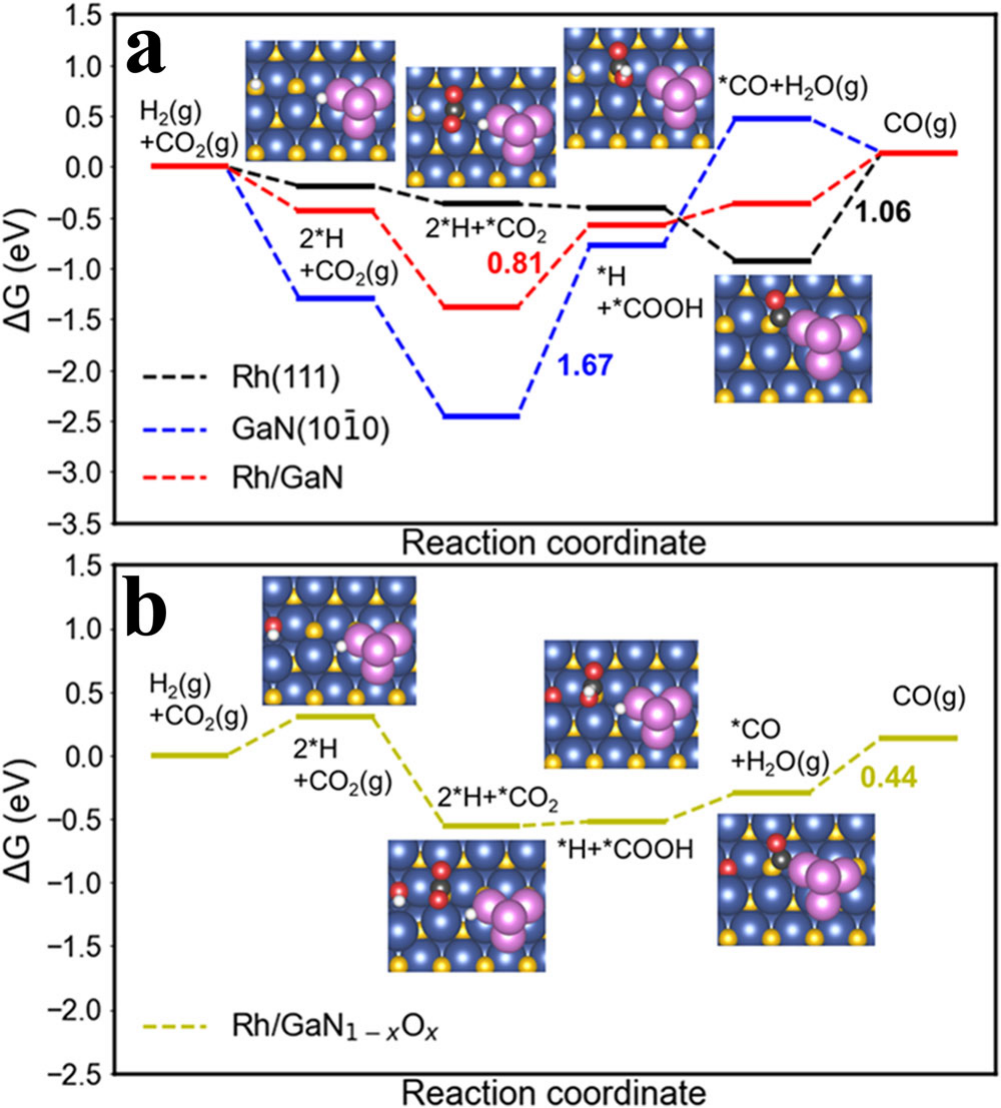
Theoretical calculations. Reaction energy profile for CO_2_ hydrogenation on **a** Rh (111), GaN (10$\bar{1}$0), Rh/GaN and **b** Rh/GaN_1–*x*_O_*x*_. The values in the figures are indicative of the energy barriers for the potential-determining step of the reaction. Ga, blue; N, yellow; Rh, purple; C, black; H, white; and O, red.

To gain more insights into the origin of the improved performance, DFT calculations were performed. Based on the previous studies^39,40^, we constructed four surfaces’ models, i.e., Rh (111), GaN (10$\bar{1}$0), Rh/GaN and Rh/GaN_1-*x*_O_*x*_, with their optimized configurations shown in Fig. S16. Compared to its original linear configuration in gas phase, apparent CO_2_ bending and C–O bond elongation are observed for all four surfaces (Table S2, Fig. 5 and Figs. S17–S18). Among the surface models structured, GaN demonstrates the largest adsorption energy (*E*_ad_), approaching to −1.71 eV, which is much larger than that of pristine Rh (−0.67 eV). It suggests the strong interaction between GaN and CO_2_, offering a solid platform for adsorbing the reactants. The substitution of oxygen atoms for nitrogen atoms by surface engineering can effectively tailor CO_2_ adsorption. According to DFT calculations, prior to annealing, the adsorption geometry of CO_2_ on Rh/GaN surface is that C atom binding to the N atom underneath and O atoms attaching to two different Ga atoms^41^. It demonstrated a relatively higher *E*_ad_ (−1.51 eV), suggesting a strong interaction between Rh/GaN and CO_2_. Notably, as presented in Fig. 5a, pristine GaN surface exhibits the strong bindings with *CO_2_ and *H, leading to the unfavored formation of *COOH with a high energy barrier of 1.67 eV. In stark contrast, after surface engineering, the adsorption geometry of CO_2_ was changed into that with C atom binding to the N atom and only one O atom attaching to the Ga atom, and the adsorption energy was correspondingly reduced to −1.38 eV. Furthermore, DFT results revealed that compared to the high *E*_ad_ (0.81 eV) on Rh/GaN surface, the *E*_ad_ of key intermediate (*COOH) formed on Rh/GaN_1–*x*_O_*x*_ was significantly reduced, indicating that the weakened CO_2_ adsorption on the catalyst was favored for the formation of *COOH. Moreover, the decoration of Rh further reduced the interaction between GaN and *CO_2_, and then facilitates the subsequent formation of *COOH (Fig. 5b). Consequently, the potential-determining step was switched from *COOH formation over Rh/GaN toward *CO desorption over Rh/GaN_1–*x*_O_*x*_. In addition, by temperature programmed desorption technique, surface engineering was found to favor the desorption of *CO from the catalyst surface (Fig. 4a), leading to a reduced *CO desorption energy barrier of 0.44 eV over Rh/GaN_1–*x*_O_*x*_-250 (Fig. 5b), thus giving rise to superior CO activity. Overall, it is found that the change in the Gibbs free energy of the reaction is as positive as 0.14 eV, indicating that CO_2_ hydrogenation to CO is an endergonic process. The spectroscopic and theoretical results above essentially show that GaN_1–*x*_O_*x*_ and Rh work synergistically to reduce the energy barrier for accelerating CO_2_ hydrogenation, which is in good agreement with the measured activity.

### Proposed mechanism

**Fig. 6:**
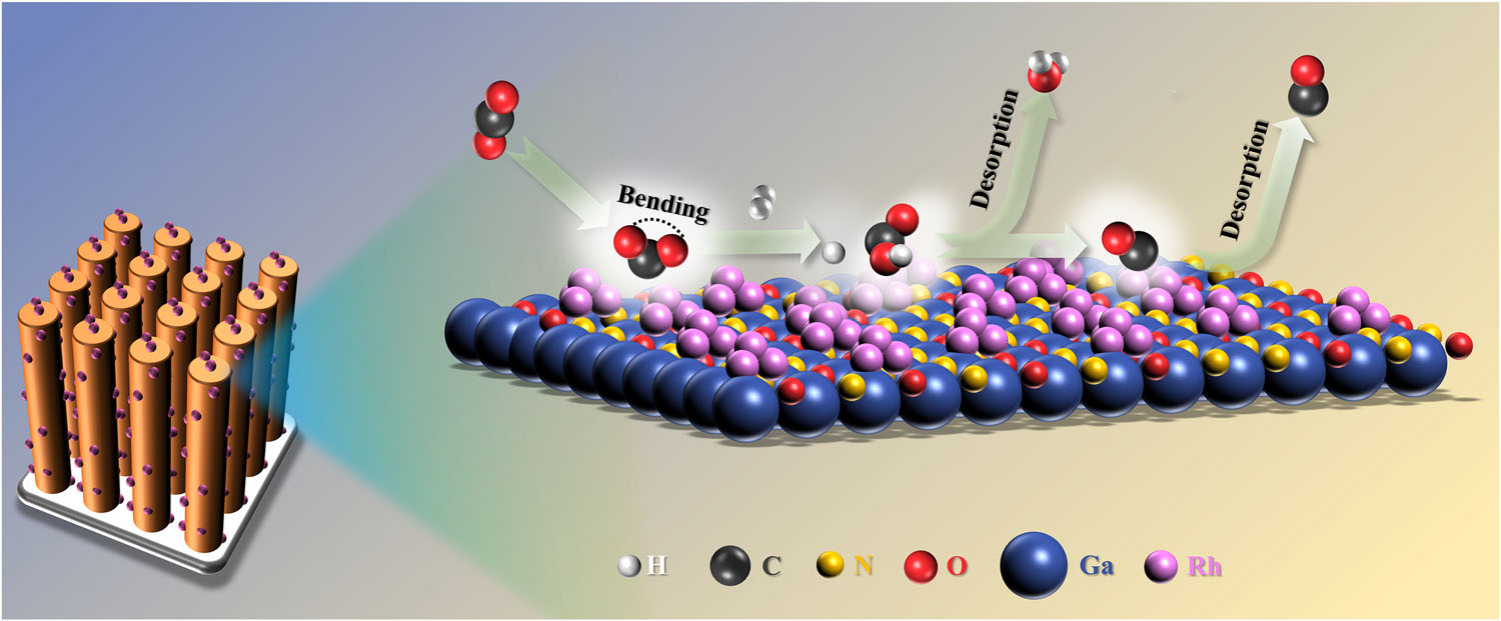
Catalytic mechanism. Schematic illustration of CO_2_ hydrogenation mechanism on the interface of Rh/GaN_1–*x*_O_*x*_.

Based on the theoretical and experimental results above, a reaction mechanism was proposed (Fig. 6). In this design, GaN_1–*x*_O_*x*_ presents an ideal platform for anchoring cocatalysts with a high density of active sites, benefitting from the well-defined 1D morphology and high surface area. The unique morphology of GaN_1–*x*_O_*x*_ nanowire arrays also facilitated the accessibility of the reactants to the active sites. On the other hand, Rh species acted as the catalytic centers for activating the inert CO_2_ molecule. Herein, it is worth noting that the interaction between active sites and CO_2_ and CO could be optimized by engineering the platform. Therefore, Rh species and GaN_1–*x*_O_*x*_ worked in synergy to promote the reaction by reducing the reaction energy barrier and accelerating the desorption of CO. In collaboration with a suitable cocatalyst of Rh, the linear CO_2_ molecule can be effectively adsorbed and deformed with significant adsorption and deformation energies, which is highly favored for the subsequent hydrogenation. When the reaction started, the adsorbed CO_2_ and H_2_ were first activated to *CO_2_ and *H. Subsequently, *CO_2_ hydrogenation reaction occurred on GaN surface in the presence of active *H species to form the key intermediate (*COOH). Finally, *COOH was reduced on Rh NPs to form *CO. The formed *CO was desorbed from the catalyst surface, which was accompanied by the formation of H_2_O. However, the strong interaction between GaN and CO_2_ results in a relatively high energy barrier during the process of *COOH formation, which limits the activity to some extent. By substituting N atom with O atom via annealing, the surface-engineered GaN_1–*x*_O_*x*_ weakens the adsorption of *CO_2_ and H*, facilitating the generation of *COOH with a significantly reduced energy barrier (Fig. 5b). Accordingly, the potential-determining step is accordingly switched from *COOH formation over Rh/GaN toward *CO desorption over Rh/GaN_1-*x*_O_*x*_ (Figs. 4a and 5). As a consequence, profiting from the synergistic effect of Rh NPs and GaN_1-*x*_O_*x*_, the evolution of CO is obviously enhanced. The discussions above validating the viability of promoting CO_2_ hydrogenation via surface engineering of the revolutionary GaN support in collaboration with Rh.

## Conclusions

In summary, in a combination of state-of-the-art molecular beam epitaxy technology with air annealing, a tunable platform of GaN_1–*x*_O_*x*_ NWs was engineered as suitable scaffolds to immobilize Rh nanoparticles for carbon dioxide hydrogenation. Both experimental and theoretical studies revealed that GaN_1–*x*_O_*x*_ worked in synergy with Rh to achieve a significantly reduced energy barrier. The potential-determining step was accordingly switched from *COOH formation over Rh/GaN toward *CO desorption over Rh/GaN_1–*x*_O_*x*_, thus resulting in the notably enhanced CO activity. A high CO activity of 127 mmol·g_cat_^−1^·h^−1^ is achieved with high selectivity of >94% at 290 °C under atmospheric pressure, vastly outperforming the commercial Rh/Al_2_O_3_. What is more, benefiting from the high cocatalyst dispersion, the architecture has enabled the achievement of a decent turnover frequency of 270 mol CO per mol Rh per hour with a total turnover number of 2616 mol CO per mol Rh. This work demonstrates a viable strategy of promoting CO_2_ hydrogenation via surface engineering of an advanced support, in collaboration with a suitable cocatalyst.

## Methods

### MBE growth

Pristine GaN NWs have been grown via radio frequency plasma-assisted molecular beam epitaxy (MBE) on 4-inch Si (111) wafer. Before nanowire growth, Si wafer was degassed under ultrahigh vacuum at 900 °C and nitridation at 800 °C. Subsequently, under constant nitrogen-rich flux, undoped GaN NWs bases with a height of about 400 nm were first grown at the set temperature, then, the Mg-doped GaN upper parts were grown with multi-steps Mg cell temperatures.

### The immobilization of Rh species

The deposition of Rh NPs was carried out in a sealed Pyrex chamber with a quartz lid through a photo-deposition process. Methanol aqueous solution with volume ratio of 1:5 (MeOH:H_2_O) and a certain volume Rh precursor (Na_3_RhCl_6_·XH_2_O) were added into the chamber. Then the chamber was filled with Ar and irradiated with 300 Xe lamp for 30 min to immobilize Rh NPs on the surface of GaN NWs, denoted by Rh/GaN. Rh/GaN_1–*x*_O_*x*_ was prepared by the same method as above except annealing the GaN surface in air for 1 h at various temperatures (150, 200, and 250 °C) before the immobilization of Rh NPs.

### Characterization

The crystal structure of the samples was analyzed by X-ray diffraction (XRD) with a Bruker D8 Advance diffractometer (with Cu Kα, at 60 kV and 80 mA). X-ray photoelectron spectroscopy (XPS) characterization was conducted by an ESCALAB 250xi non-monochromatic Al anodes; and a peak of C 1s at 284.8 eV was used as an internal standard to calibrate the binding energies. Induced coupled plasma (ICP) measurements were performed using an AGILENT ICP-OES 730. High-angle annular dark field-scanning transmission electron microscopy (HAADF-STEM) images were obtained using a Thermo Fisher Scientific Talos F200X S/TEM, equipped with a Super-X EDS detector and operated at 200 kV. Transmission electron microcopy (TEM) images of the pre-synthesized samples were captured at 200 kV with a JEOL 2100 F microscope. Scanning electron microcopy (SEM) images were captured by Quattro ESEM (Thermo Fisher). Temperature Programmed Desorption (TPD) was measured by Autosorb-iQ-C chemisorption analyzer (Quantachrome, USA). In situ diffuse reflection Fourier transform infrared (DRFTI) was characterized by Frontier FT-IR Spectrometer, PerkinElmer, which was equipped with an MCT detector and 10-cm Demountable Gas Cell. Test methods are as follows: after vacuum degassing, the sample was pressed and vacuumed in the infrared pool for 10 min, and the sample data were collected. Then CO_2_ was injected into the infrared cell. After 15 min adsorption, CO_2_ was discharged and infrared data was collected.

### Performance evaluation

The CO_2_ hydrogenation was performed in a closed stainless-steel chamber under atmospheric pressure. Before fixed at the bottom of the stainless-steel reaction cell, the wafer-based catalysts were thoroughly washed with deionized water. After evacuation, the mixture of CO_2_ and H_2_ with various ratios was injected the chamber. The reaction was carried at the setting temperatures. Aft the reaction, the chamber was cool down to room temperature. The gaseous and liquidous products were analyzed by a gas chromatograph (GC-9080, Sun) equipped with a flame ionization detector (FID) detector and a thermal conductivity detector (TCD). Calculation of CO evolution rate, turnover frequency (TOF) and turnover number (TON) based on the following equation.

$${{{{{\rm{CO}}}}}}\; {{{{{\rm{evolution}}}}}}\; {{{{{\rm{rate}}}}}}=\frac{{{{\mbox{CO}}}} \, {{{\mbox{yield}}}} \, {{{\mbox{per}}}} \, {{{\mbox{unit}}}} \, {{{\mbox{area}}}} \, {{{\mbox{of}}}} \, {{{\mbox{catalyst}}}} \, {{{\mbox{per}}}} \, {{{\mbox{hour}}}}}{{{{{\mbox{Mass}}}} \, {{{\mbox{of}}}} \, {{{\mbox{catalyst}}}} \, {{{\mbox{per}}}} \, {{{\mbox{unit}}}} \, {{{\mbox{area}}}}}}$$

$${{{{{\rm{TOF}}}}}}=\frac{{{{\mbox{CO}}}} \, {{{\mbox{evolution}}}} \, {{{\mbox{rate}}}}}{{{{\mbox{Content}}}} \, {{{\mbox{of}}}} \, {{{\mbox{immobilized}}}} \, {{{\mbox{Rh}}}} \, {{{\mbox{NPs}}}}} \, {{{{{\rm{TON}}}}}}=\frac{{{{\mbox{Yield}}}} \, {{{\mbox{of}}}} \, {{{\mbox{CO}}}}}{{{{\mbox{Content}}}} \, {{{\mbox{of}}}} \, {{{\mbox{immobilized}}}} \, {{{\mbox{Rh}}}} \, {{{\mbox{NPs}}}}}$$

### Computational methods

All calculations were based on the ab initio spin-polarized density functional theory (DFT) calculations^42^ employing the Vienna Ab Initio Simulation Package (VASP)^43,44^. The projector-augmented wave (PAW) method was used to describe the interactions of valence electrons with the ionic cores^45^, and Perdew-Burke-Ernzerhof (PBE) functional was used to describe the exchange-correlation of the Kohn–Sham equation^46^. Valence electron functions are expanded in plane waves with a kinetic energy cutoff of 500 eV, and a 2 × 2 × 1 k-point grid was used during structural relaxation for all slabs until the energy differences converged to 10^−4 ^eV and the Hellmann-Feynman forces converged to 0.02 eV/Å. Grimme’s DFT-D3 method was applied for all calculations to include the effect of weak van der Waals (vdW) interactions^47,48^.

Rh (111), GaN (10$\bar{1}$0), Rh/GaN and Rh/GaN_1–*x*_O_*x*_ surface models were adopted to model Rh, pristine GaN, Rh/GaN and Rh/GaN_1–*x*_O_*x*_ catalysts (Fig. S16). Rh (111) and GaN (10$\bar{1}$0) slabs were constructed in 6 × 6 arrangement, and Rh/GaN was constructed by depositing a cluster of 4 Rh atoms on GaN (10$\bar{1}$0) surface. Rh/GaN_1–*x*_O_*x*_ model was created by replacing one nitrogen atom on the Rh/GaN surface with one oxygen atom. For all models including GaN slab, the dangling bonds at the bottom of the Ga and N atoms were passivated with pseudo hydrogen atoms having valence charges of 4/3 and 3/4, respectively. During structural relaxation, the atoms in the topmost two layers in Rh (111) and the topmost three layers in GaN and GaN_1–*x*_O_*x*_ were allowed to relax, while the rest of the atoms were fixed in their equilibrium bulk positions. A vacuum spacing of at least 12 Å along the direction normal to the slab surface was used to eliminate image interaction across the periodic boundary.

The computational hydrogen electrode (CHE) model was used to calculate the free energies of CO_2_ hydrogenation^49^. The Gibbs free energy of adsorption ∆G was calculated as:

$$\triangle G={E}_{{{{{{\rm{ad}}}}}}}+\triangle {{{{{\rm{ZPE}}}}}}+\int \triangle {C}_{p}{dT}-T\triangle S$$

Here, *E*_ad_ is the calculated adsorption energy of an adsorbate on a catalyst surface. ΔZPE, Δ*C*_*p*_ and Δ*S* represent the change in zero-point energy, heat capacity and entropy, respectively. The temperature *T* was set as the room temperature, 298.15 K.

1 $${{{{{{\rm{H}}}}}}}_{2}({{{{{\rm{g}}}}}})+{2}^{\ast }\to {2}^{\ast }{{{{{\rm{H}}}}}}$$

2 $${{{{{{\rm{CO}}}}}}}_{2}({{{{{\rm{g}}}}}})+{}^{\ast }\to {}^{\ast }{{{{{{\rm{CO}}}}}}}_{2}$$

3 $${}^{\ast }{{{{{{\rm{CO}}}}}}}_{2}+{}^{\ast }{{{{{\rm{H}}}}}}\to {}^{\ast }{{{{{\rm{COOH}}}}}}{+}^{\ast }$$

4 $${}^{\ast }{{{{{\rm{COOH}}}}}}+{}^{\ast }{{{{{\rm{H}}}}}}\to {}^{\ast }{{{{{\rm{CO}}}}}}+{{{{{{\rm{H}}}}}}}_{2}{{{{{\rm{O}}}}}}({{{{{\rm{l}}}}}})+{}^{\ast }$$

5 $${}^{\ast }{{{{{\rm{CO}}}}}}\to {}^{\ast }+{{{{{\rm{CO}}}}}}({{{{{\rm{g}}}}}})$$

## Supplementary information

Supplementary Information

## Data Availability

All the data are available in the manuscript and supplementary materials.
