# Supplementary material for: Oxynitride-surface engineering of rhodium-decorated gallium nitride for efficient thermocatalytic hydrogenation of carbon dioxide to carbon monoxide
Source: Commun Chem. 2022 Sep 6;5:107. doi: 10.1038/s42004-022-00728-x (PMC9814893; doi:10.1038/s42004-022-00728-x)
Supplement: Supplementary file 1 — Supplementary Information [file 42004_2022_728_MOESM1_ESM.pdf]

# Oxynitride-surface engineering of rhodium-decorated gallium nitride for efficient thermocatalytic hydrogenation of carbon dioxide to carbon monoxide

Jinglin Li[1+], Bowen Sheng[2+], Yiqing Chen[3+], Sharif Md. Sadaf[4], Jiajia Yang[2], Ping Wang[2], Hu Pan[1], Tao Ma[5], Lei Zhu[1], Jun Song[3*], He Lin[1], Xinqiang Wang[2,6,7*], Zhen Huang[1*], Baowen Zhou[1*].

1. Key Laboratory for Power Machinery and Engineering of Ministry of Education, School of Mechanical Engineering, Shanghai Jiao Tong University, 800 Dongchuan Road, Shanghai 200240, China.
2. State Key Laboratory of Artificial Microstructure and Mesoscopic Physics, School of Physics, Nano-Optoelectronics Frontier Center of Ministry of Education (NFC-MOE), Peking University, Beijing 10087, China.
3. Department of Mining and Materials Engineering, McGill University, 3610 University Street, Montreal, QC H3A0C9, Canada
4. Centre Energie, Matériaux et Télécommunications, Institut National de la Recherche Scientifique (INRS)-Université du Québec, 1650 Boulevard Lionel-Boulet, Varennes, Quebec J3X1S2, Canada.
5. Michigan Center for Materials and Characterization, University of Michigan, 2800 Plymouth Rd, Ann Arbor, MI 48109, USA
6. Peking University Yangtze Delta Institute of Optoelectronics, Nantong, Jiangsu 226010, China
7. Collaborative Innovation Center of Quantum Matter, School of Physics, Peking University, Beijing 100871, China.

* Corresponding authors Email: jun.song2@mcgill.ca; wangshi@pku.edu.cn; z-huang@sjtu.edu.cn; zhoubw@sjtu.edu.cn

+ These authors contributed equally to this work.

**Table S1** Performance comparison of $CO_2$ hydrogenation toward CO catalyzed by state-of-the-art catalytic architectures.

| Catalyst | T (°C) | P (MPa) | CO rate ($mmol \cdot g_{cat}^{-1} \cdot h^{-1}$) | CO Select. (%) | Ref. |
|---|---|---|---|---|---|
| $Rh/GaN_xO_{1-x}/Si$ | 260 | 0.1 | 106.4 | 94 | This work |
| $Rh/GaN_xO_{1-x}/Si$ | 290 | 0.1 | 127 | 94 | This work |
| $CoZrO_x$ | 340 | 3 | 13.7 | 97 | Ref. (1) |
| $Ni_3$-$Fe_9/ZrO$ | 400 | 0.1 | 23.0 | 96 | Ref. (2) |
| Rh/S-1 | 300 | 1 | 0.02 | 86 | Ref. (3) |
| 10% Fe-$sMoS_2$ | 500 | 1 | 56.5 | 99 | Ref. (4) |
| Pt-Co/$TiO_2$ | 300 | - | 41.0 | 99 | Ref. (5) |

**Table S2** Geometry parameters, adsorption energies and deformation energies of $CO_2$ adsorption on Rh (111), GaN ($10\bar{1}0$), Rh/GaN and Rh/$GaN_{1-x}O_x$ compared to gas phase $CO_2$.

| Species (*$CO_2$) | Bond length (Å) | | Angle (°) | Energy (eV) | |
|---|---|---|---|---|---|
| | C-O1 | C-O2 | ∠O1-C-O2 | $E_{ad}$ | $E_{def}$ |
| Gas phase | 1.18 | 1.18 | 180 | - | 0.00 |
| Rh (111) | 1.22 | 1.29 | 131.54 | -0.67 | 2.02 |
| GaN ($10\bar{1}0$) | 1.28 | 1.30 | 127.60 | -1.71 | 2.59 |
| Rh/GaN | 1.27 | 1.31 | 127.84 | -1.51 | 2.57 |
| Rh/$GaN_{1-x}O_x$ | 1.24 | 1.31 | 126.04 | -1.38 | 2.60 |

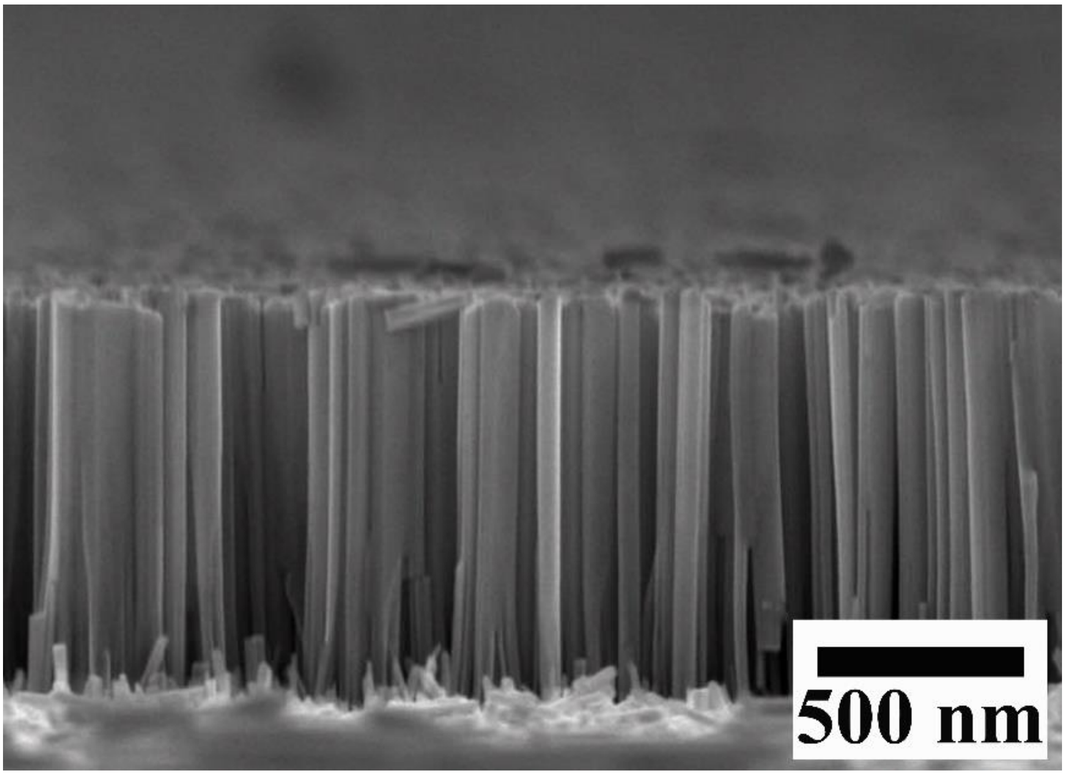


**Fig. S1** SEM image of pristine GaN NWs vertically aligned onto Si wafer.

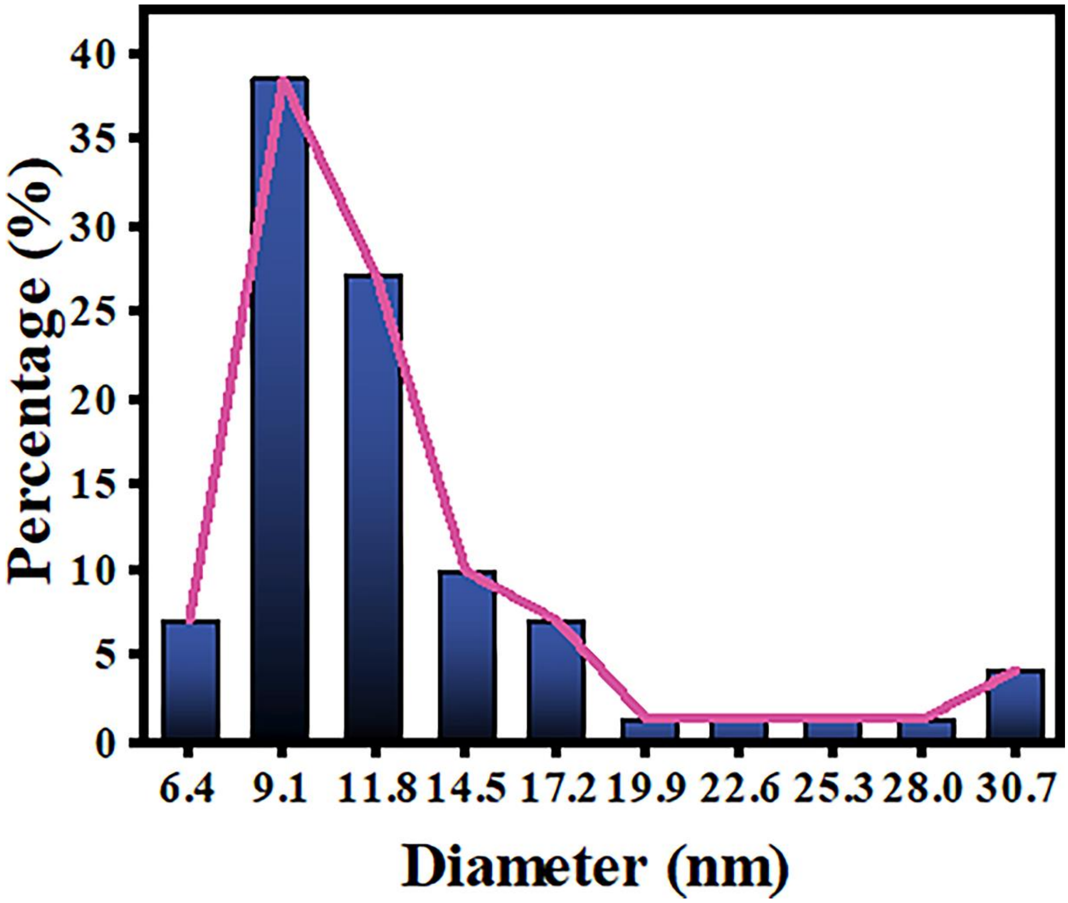


**Fig. S2** The size distribution of Rh/GaN with 70 µl Rh precursor.

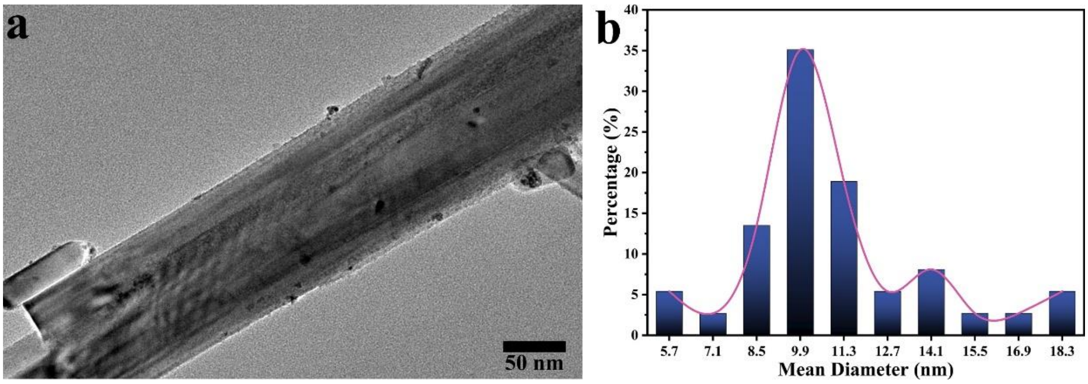


**Fig. S3** (a) TEM images and (b) Rh NPs diameters distribution of Rh/$GaN_{1-x}O_x$-250.

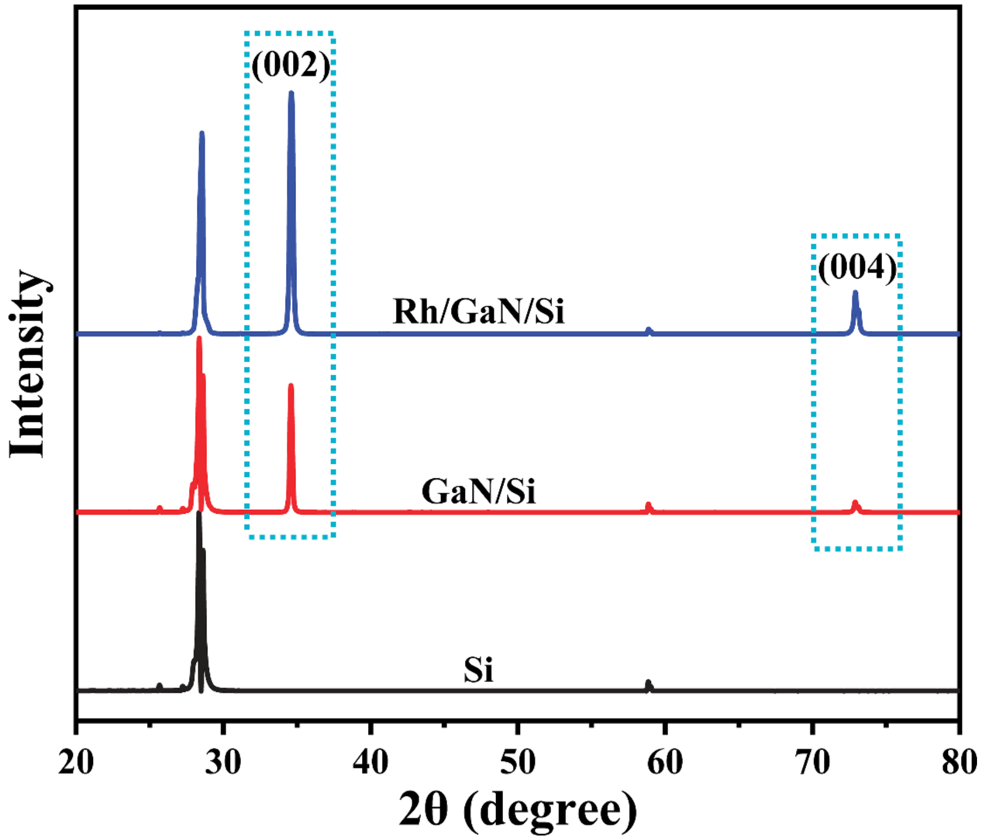


**Fig. S4** XRD patterns of Si wafer, GaN and Rh/GaN supported by Si wafer, respectively.

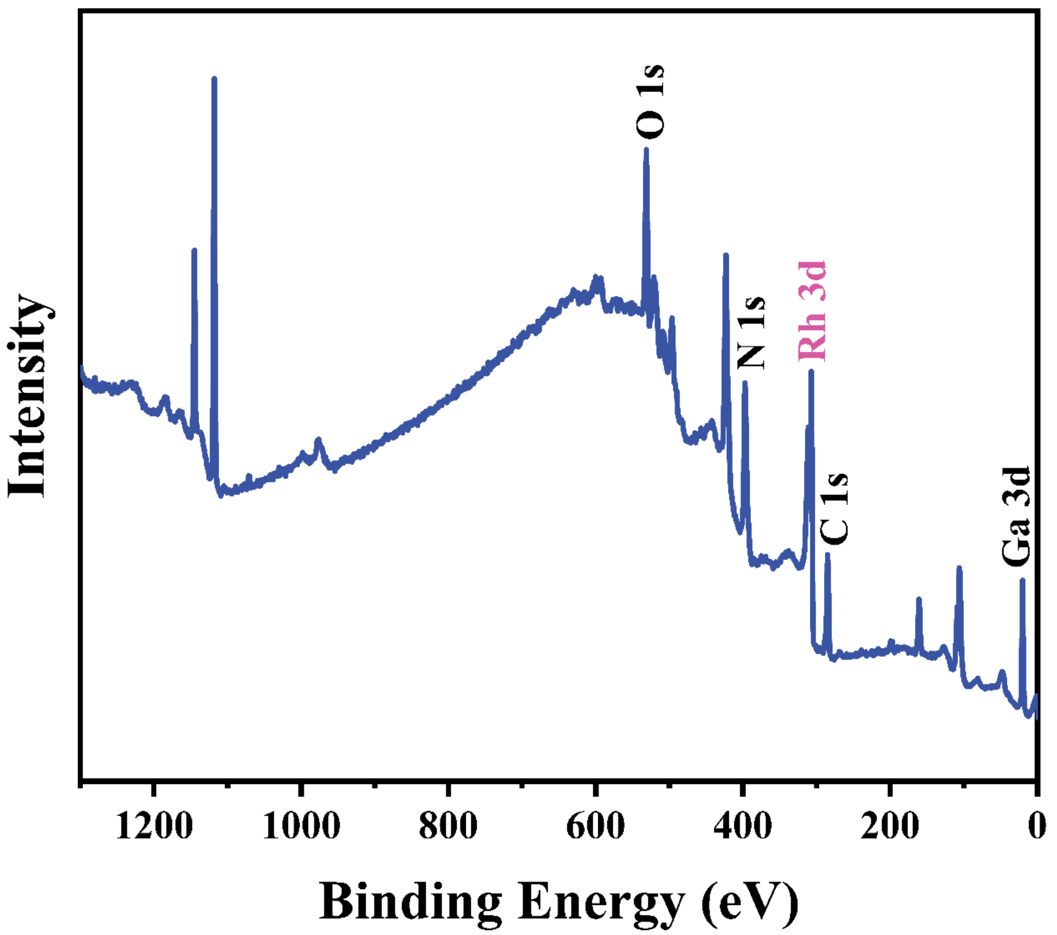


**Fig. S5** XPS survey of Rh/Ga$N_{1-x}O_x$-250 supported by Si substrate.

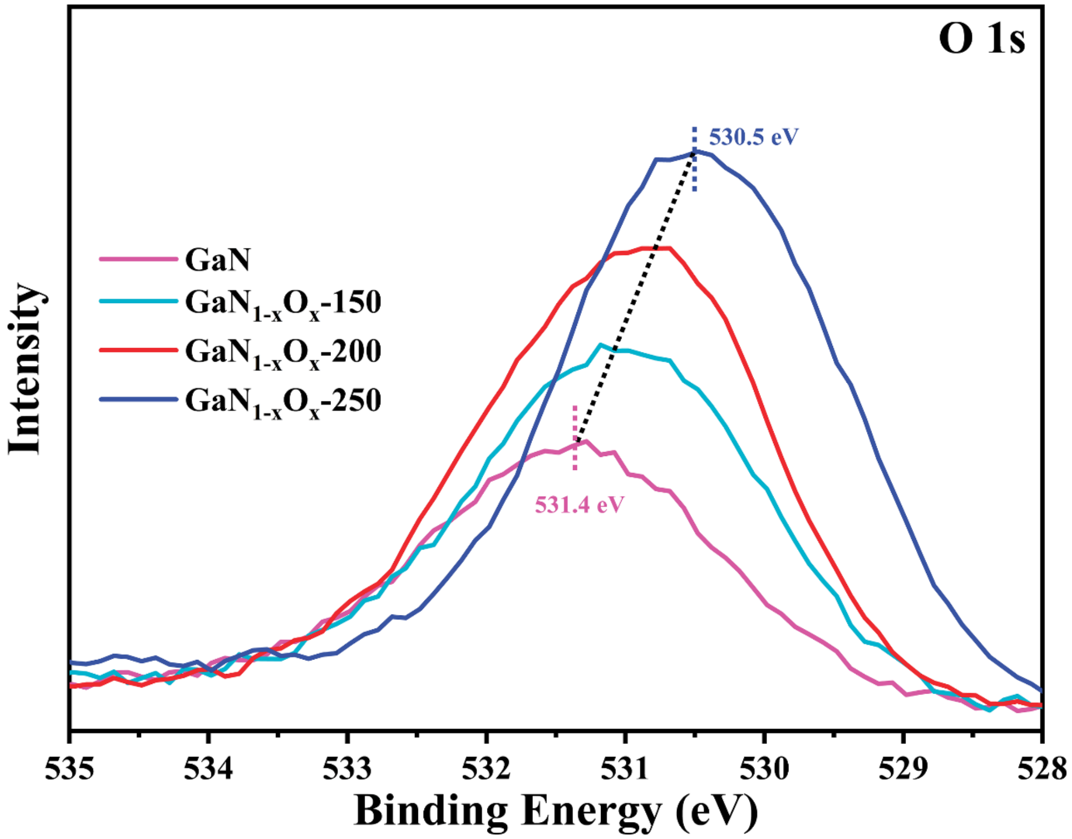


**Fig. S6** High-resolution XPS spectra of O 1s from GaN, $GaN_{1-x}O_x$-150, $GaN_{1-x}O_x$-200 and $GaN_{1-x}O_x$-250.

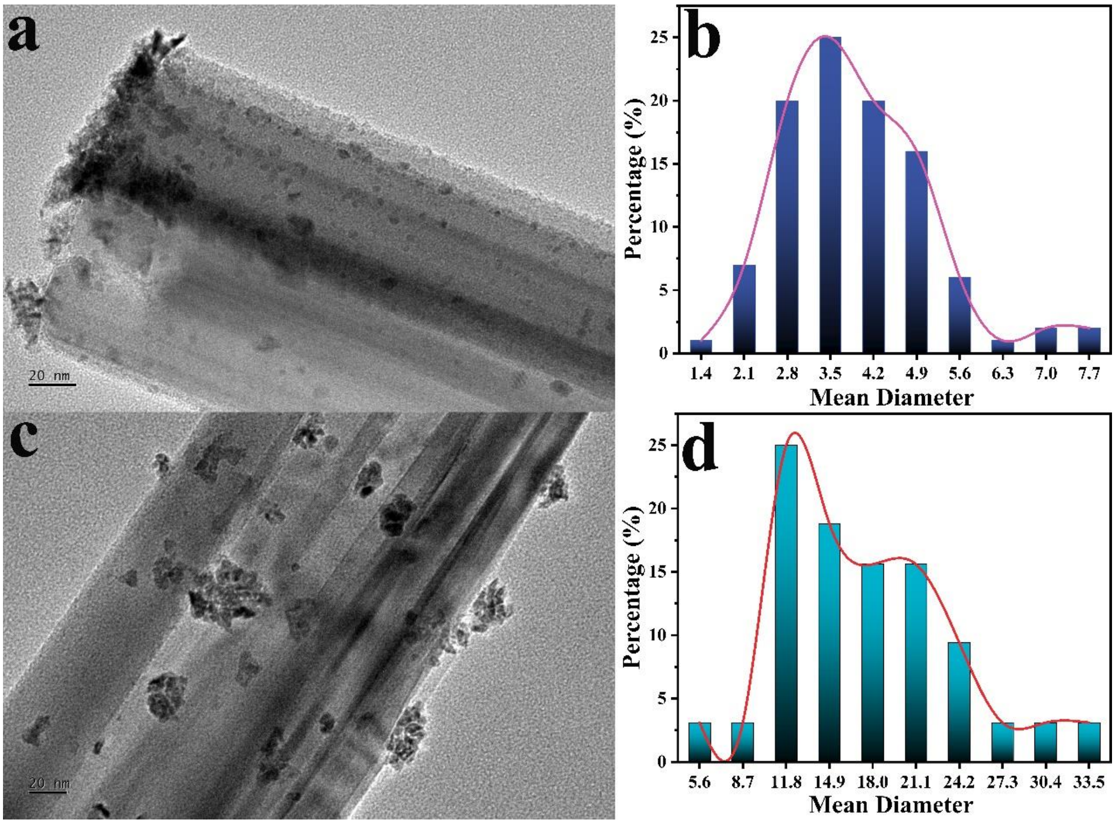


**Fig. S7 Morphology and particle size analysis of Rh/GaN with 5 and 135 µl Rh precursor.** (a), (c) TEM images and (b), (d) Rh NPs diameters distribution, respectively.

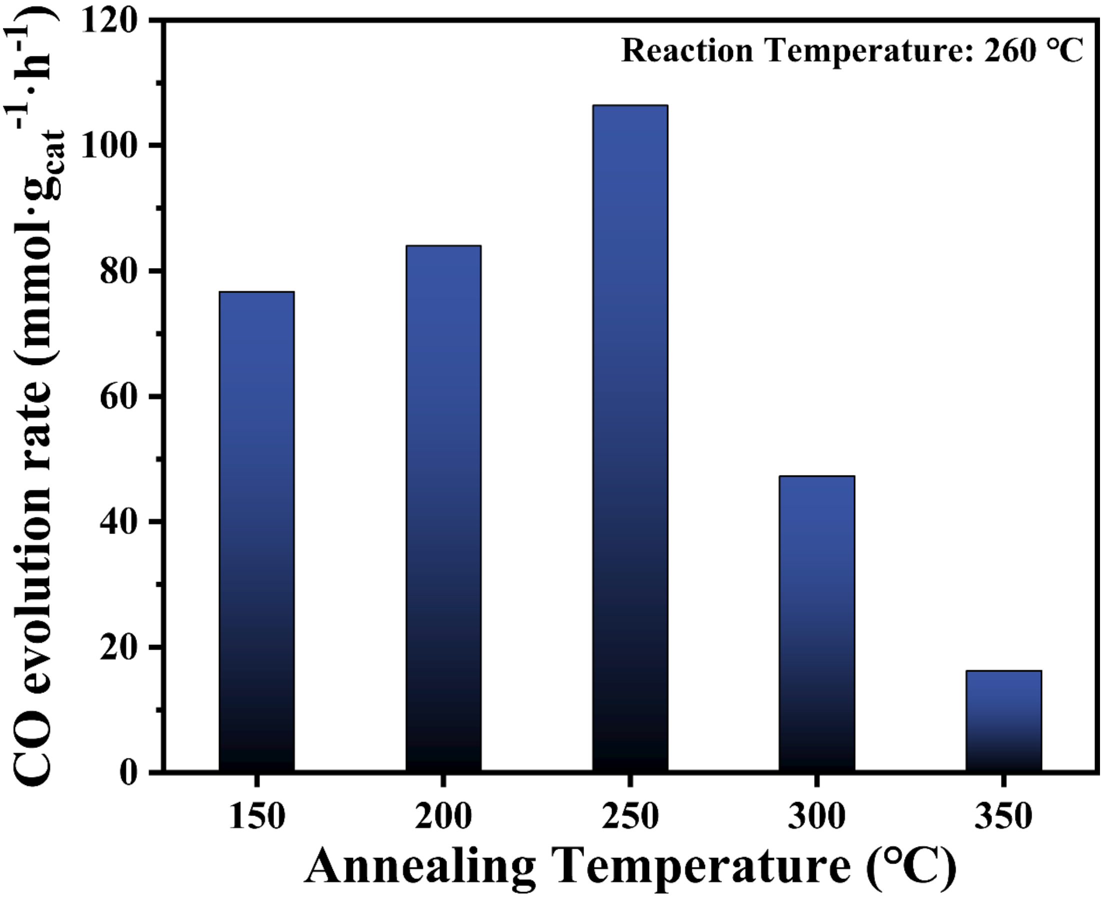


**Fig. S8** Influence of the annealing temperature on the performance.

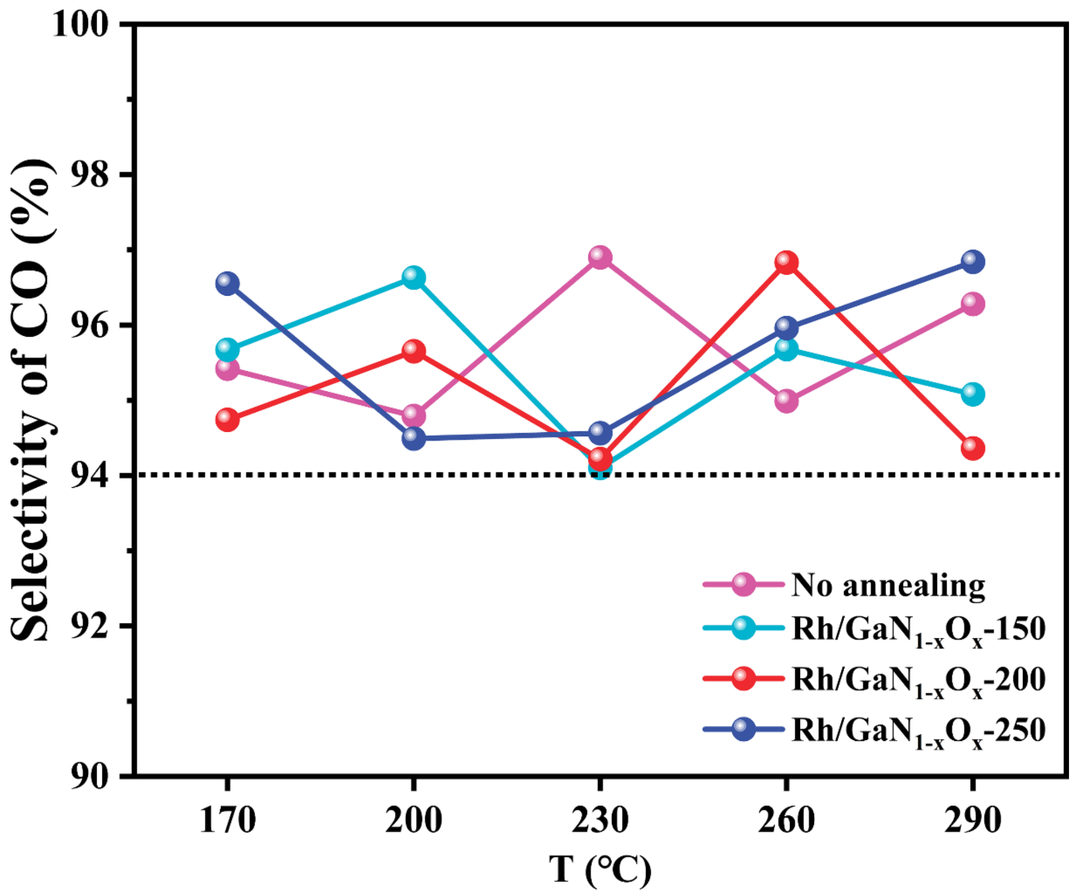


**Fig. S9** Selectivity of CO over Rh/GaN and various annealed Rh/$GaN_{1-x}O_x$ samples.

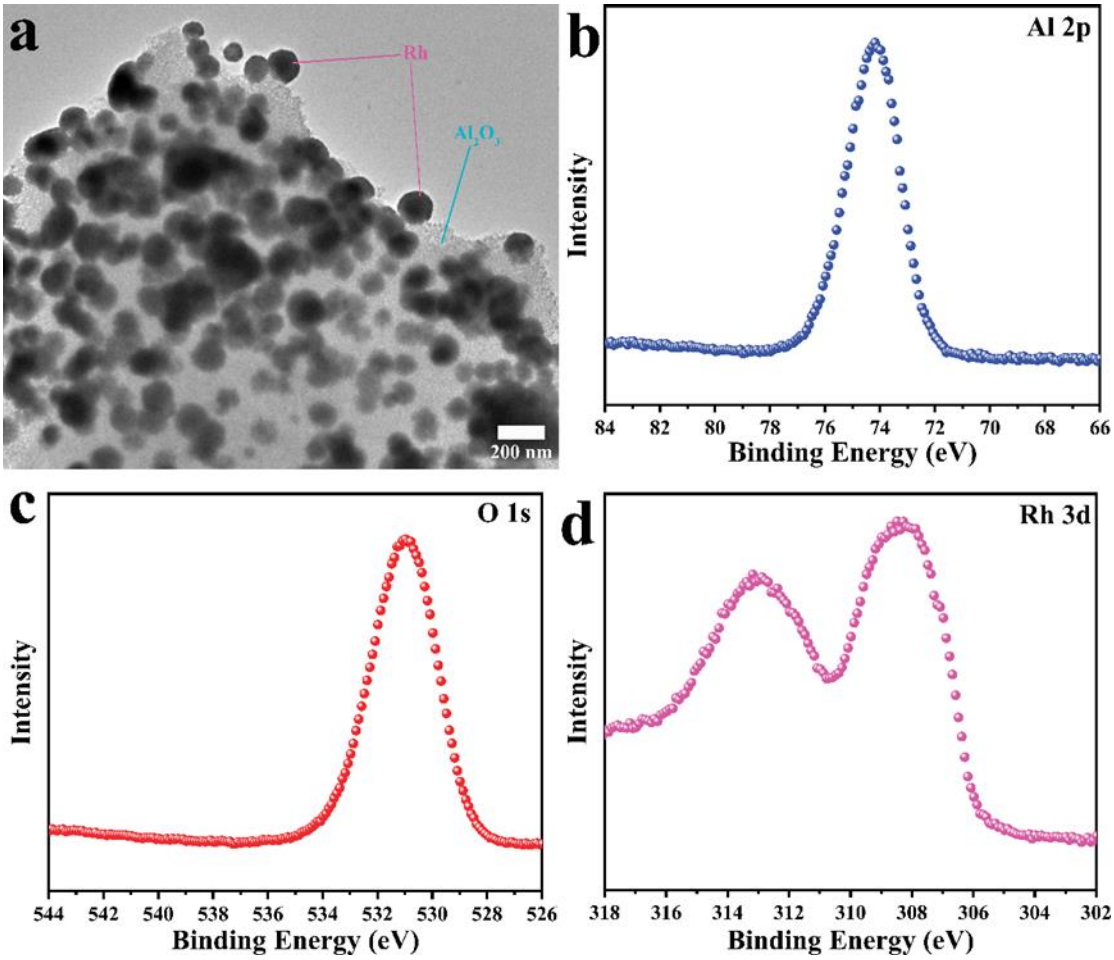


**Fig. S10** (a) TEM image and (b-d) XPS spectra of commercial Rh/$Al_2O_3$.

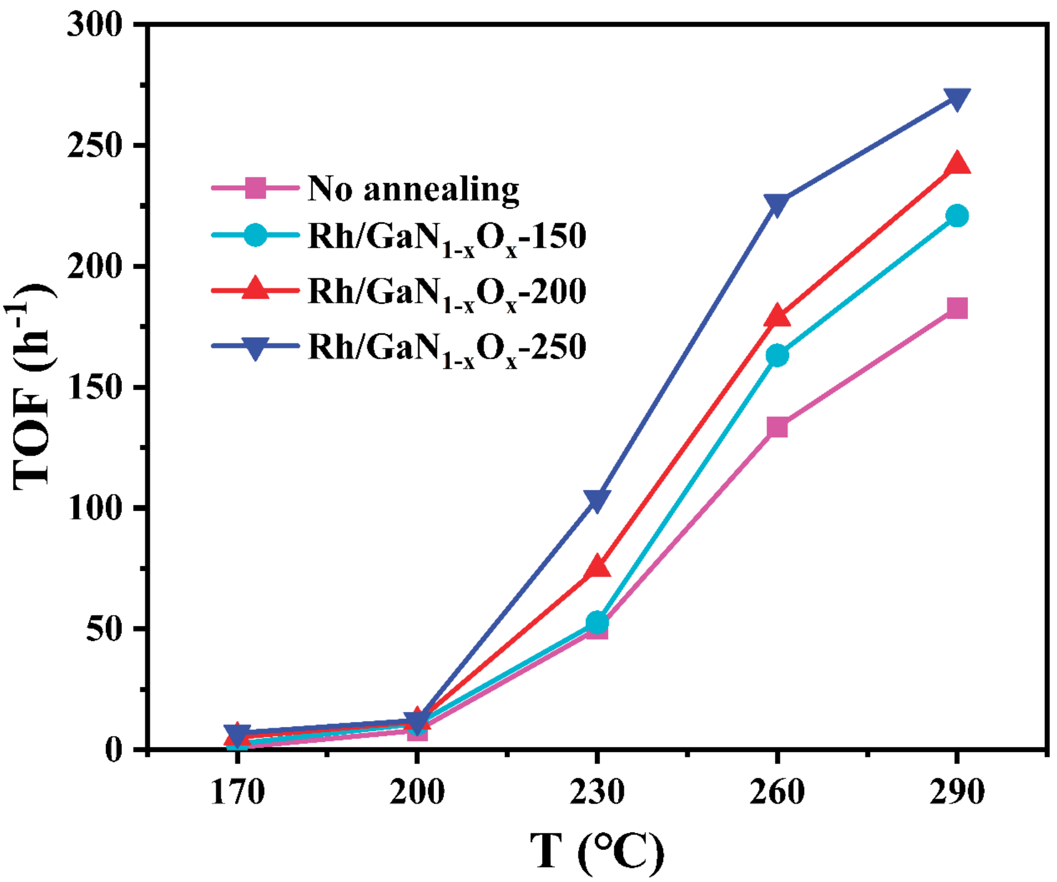


**Fig. S11** The calculated TOF of CO over Rh/GaN and various Rh/$GaN_{1-x}O_x$ under different reaction temperatures.

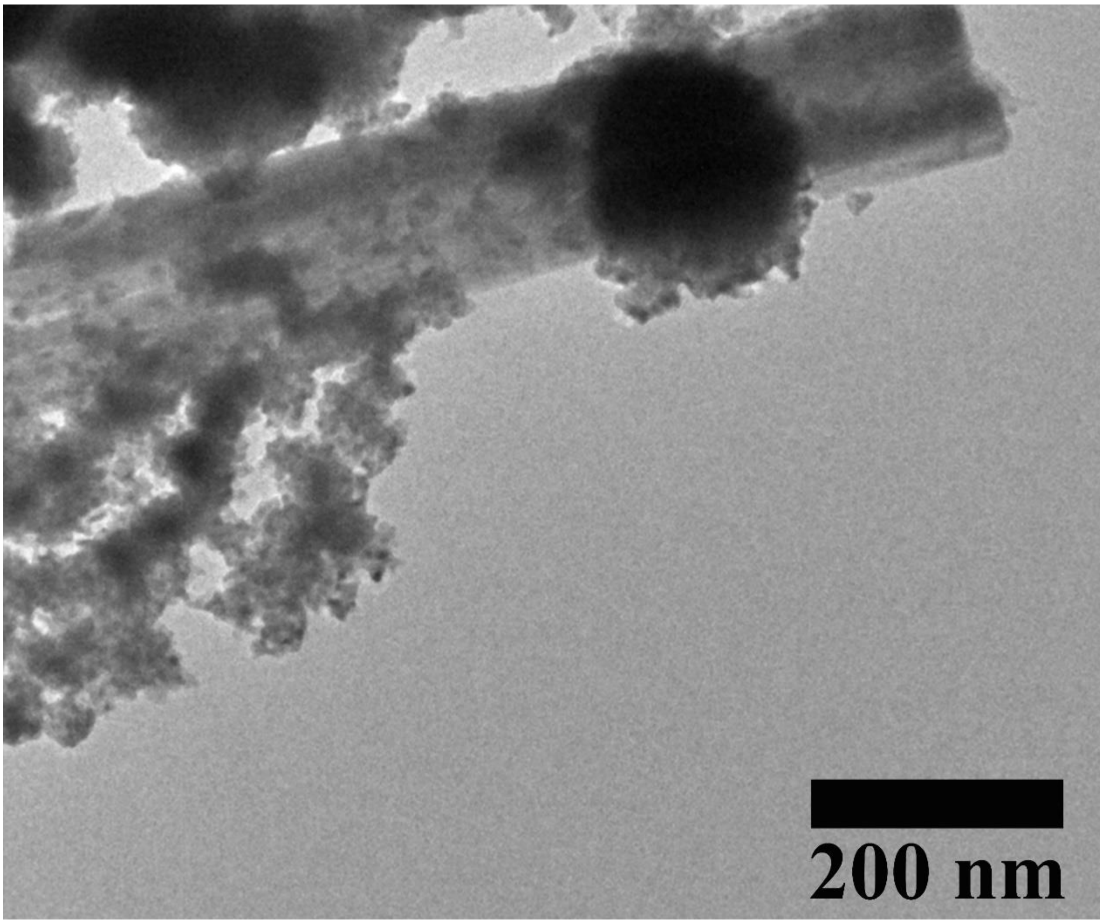


**Fig. S12** TEM image of Rh/$GaN_{1-x}O_x$ after 9 cycles of reusability testing.

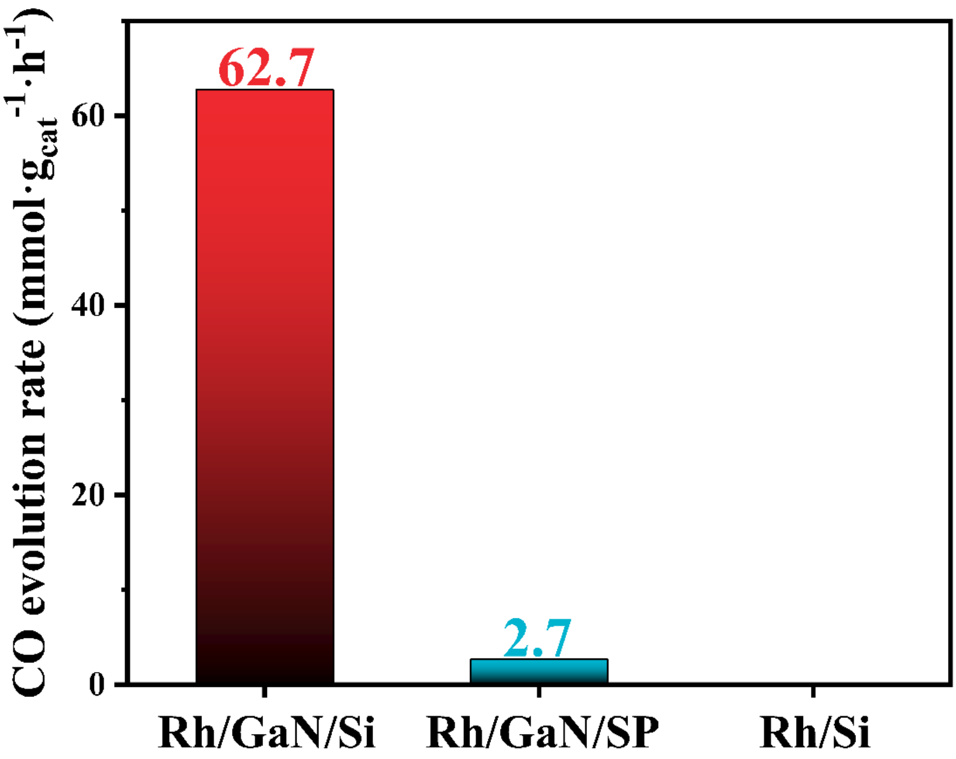


**Fig. S13** CO evolution rate over different Rh-decorated supports.

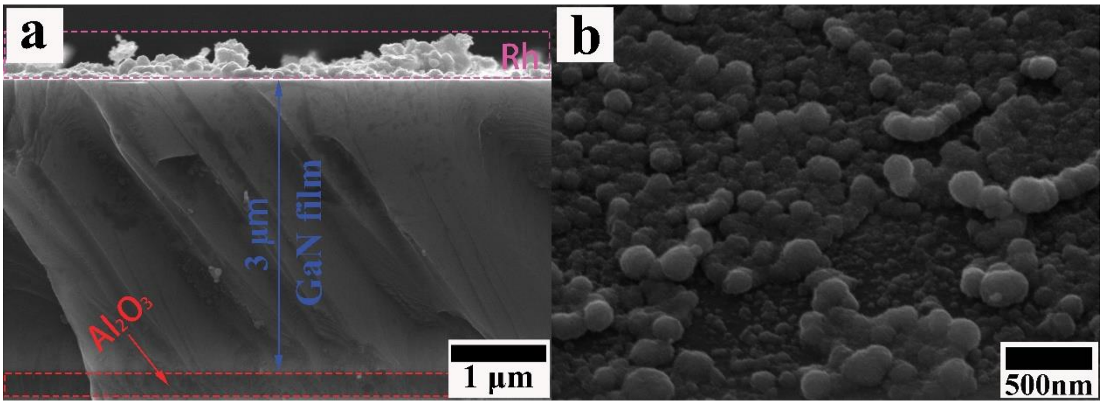

**Fig. S14** SEM images of Rh/GaN/SP. (a) side view. (b) top view.

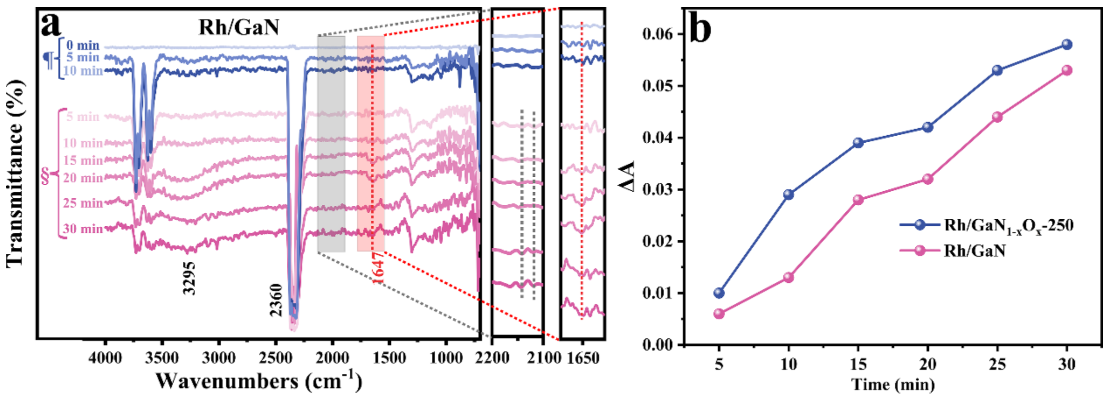


**Fig. S15** (a) *In situ* DRIFT spectra of Rh/GaN, ¶: from room temperature to 260 °C; §: keeping 260 °C. (b) Slope change of the peak intensity of *COOH derived from *in situ* DRIFT spectra in **Fig. 4b** and **S15a.**

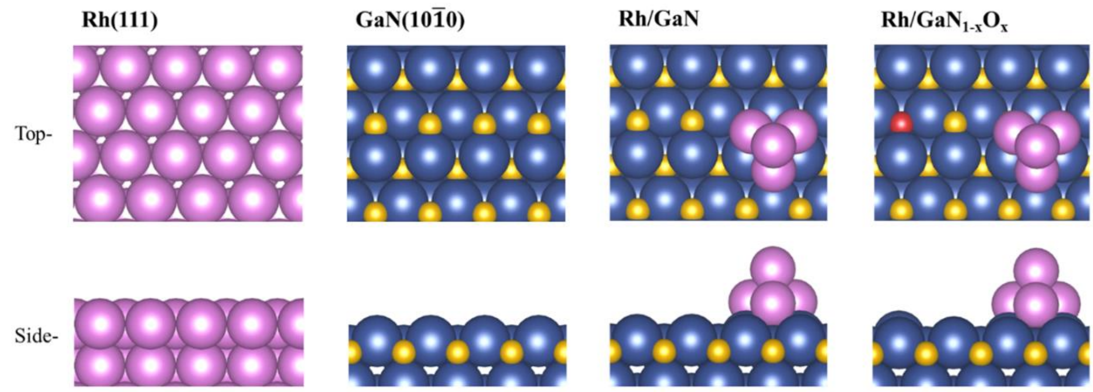


**Fig. S16** The top and side views of the optimized geometries of Rh (111), GaN (10$\bar{1}$0), Rh/GaN and Rh/$GaN_{1-x}O_x$. Ga, blue; N, yellow; Rh, purple; and O, red.

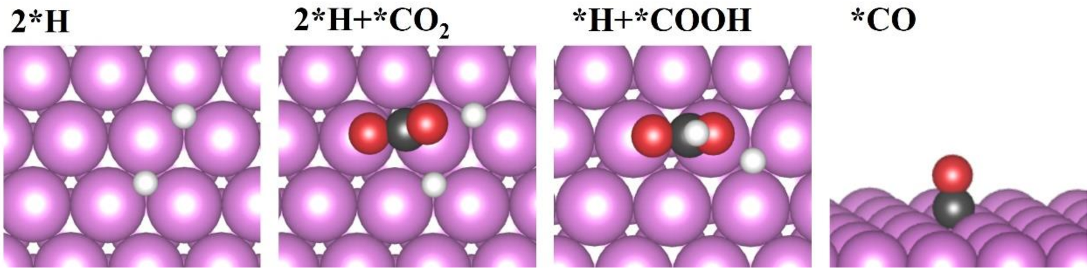


**Fig. S17** Adsorption configurations for each reaction intermediate of $CO_2$ hydrogenation on Rh (111). Rh, purple; C, black; H, white; and O, red.

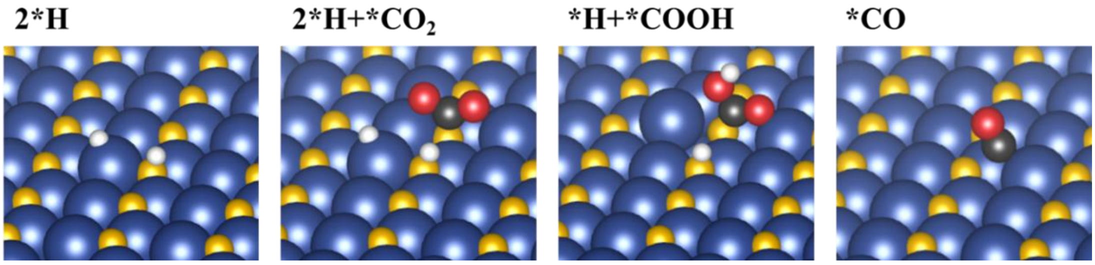


**Fig. S18** Adsorption configurations for each reaction intermediate of $CO_2$ hydrogenation on GaN $(10\bar{1}0)$. Ga, blue; N, yellow; C, black; H, white; and O, red.

## References

1. N. H. M. D. Dostagir et al. Co Single Atoms in $ZrO_2$ with Inherent Oxygen Vacancies for Selective Hydrogenation of $CO_2$ to CO. *ACS Catal.* **11**, 9450-9461 (2021).
2. B. Yan et al. Tuning $CO_2$ hydrogenation selectivity via metal-oxide interfacial sites. *J. Catal.* **374**, 60-71 (2019).
3. C. Wang et al. Product Selectivity Controlled by Nanoporous Environments in Zeolite Crystals Enveloping Rhodium Nanoparticle Catalysts for $CO_2$ Hydrogenation. *J. Am. Chem. Soc.* **141**, 8482-8488 (2019).
4. J. Zheng et al. High Loading of Transition Metal Single Atoms on Chalcogenide Catalysts. *J. Am. Chem. Soc.* **143**, 7979-7990 (2021).
5. S. Kattel et al. $CO_2$ Hydrogenation over Oxide-Supported PtCo Catalysts: The Role of the Oxide Support in Determining the Product Selectivity. *Angew. Chem. Int. Ed.* **55**, 7968-7973 (2016).
